# Rationale of the Spanish FRAX model in decision-making for predicting osteoporotic fractures: an update of FRIDEX cohort of Spanish women

**DOI:** 10.1186/s12891-016-1096-6

**Published:** 2016-06-17

**Authors:** Rafael Azagra, Marta Zwart, Gloria Encabo, Amada Aguyé, Juan Carlos Martin-Sánchez, Nuria Puchol-Ruiz, Paula Gabriel-Escoda, Sergio Ortiz-Alinque, Emilio Gené, Milagros Iglesias, David Moriña, Miguel Angel Diaz-Herrera, Mireia Utzet, Josep Maria Manresa, On behalf of GROIMAP study group

**Affiliations:** Department of Medicine, Universitat Autònoma de Barcelona, ps/Vall de Hebron 119, 08135 Barcelona, Spain; Health Center Badia del Valles, Institut Català de la Salut, GROIMAP-USR MN-IDIAP Jordi Gol, c/Bética s/n, 08214 Badia del Vallés, Barcelona Spain; QuironSalud-Hospital General de Catalunya, Universitat Internacional de Catalunya, c/Josep Trueta s/n, 08195 Sant Cugat del Vallès, Barcelona Spain; Health Center Can Gibert del Plà (ICS), Institut Català de la Salut, GROIMAP-USR Girona-IDIAP Jordi Gol, c/San Sebastian 9, 17005 Girona, Spain; Department of Nuclear Medicine, Valle de Hebron Hospital, Institut Català de la Salut, Ps/Valle de Hebron 119-129, 08035 Barcelona, Spain; Health Center Granollers-Centre, Institut Català de la Salut, c/Museu 19, 08400 Granollers, Barcelona Spain; Department of Basic Sciences, Biostatistics Unit, Universitat Internacional de Catalunya, c/Josep Trueta s/n, 08195 Sant Cugat del Valles, Barcelona Spain; Health Center Barberà del Vallès, Institut Català de la Salut, GROIMAP-USR MN-IDIAP Jordi Gol, c/Verge de l’Assumpció s/n, 08210 Barberà del Vallès, Barcelona Spain; Health Center Canaletes, Institut Català de la Salut, GROIMAP-USR MN-IDIAP Jordi Gol, c/Ps d’Horta 17, 08290 Cerdanyola del Vallès, Barcelona Spain; Department of Medicine, Universitat Internacional de Catalunya, c/Josep Trueta s/n, 08195 Sant Cugat del Valles, Barcelona Spain; Urgencies Service, Hospital of Sabadell, Corporació Sanitaria i Universitaria Parc Tauli, Parc Tauli s/n, 08208, Sabadell, Barcelona Spain; Unit of Infections and Cancer (UNIC), Cancer Epidemiology Research Program (CERP), Catalan Institute of Oncology (ICO)-IDIBELL, Av Gran Via, 199-203, 08908 L’Hospitalet de Llobregat, Barcelona Spain; Departament d’Economia i Història Econòmica, Grups de Recerca d’Àfrica i Amèrica Llatines (GRAAL), Unitat de Fonaments de l’Anàlisi Econòmica, Universitat Autònoma de Barcelona, c/Emprius 2, 08202 Sabadell, Barcelona Spain; Health Center Cornellà 2 (Sant Ildefons), Institut Català de la Salut, GROIMAP-USR MN-IDIAP Jordi Gol, c/Republica Argentina s/n, 08940 Cornellá, Barcelona Spain; Biostatistics Unit, CUPESSE European Project, Universitat Pompeu Fabra, Ed Jaume I-Campus Ciutadella, 08003 Barcelona, Spain; Unitat Supor Recerca Metropolitana Nord, IDIAP Jordi Gol, ctra de Barcelona 473, 08204 Sabadell, Barcelona Spain; Department of Nursing, Universitat Autònoma de Barcelona, avda Can Domenech s/n, 08193 Cerdanyola del Valles, Barcelona Spain

**Keywords:** Osteoporosis, Fracture, FRAX, FRIDEX, Study cohort, Algorithm

## Abstract

**Background:**

The FRAX® tool estimates the risk of a fragility fracture among the population and many countries have been evaluating its performance among their populations since its creation in 2007.

The purpose of this study is to update the first FRIDEX cohort analysis comparing FRAX with the bone mineral density (BMD) model, and its predictive abilities.

**Methods:**

The discriminatory ability of the FRAX was assessed using the ‘area under curve’ of the receiver operating characteristic (AUC-ROC). Predictive ability was assessed by comparing estimated risk fractures with incidence fractures after a 10-year follow up period.

**Results:**

One thousand three hundred eight women ≥ 40 and ≤ 90 years followed up during a 10-year period. The AUC for major osteoporotic fractures using FRAX without DXA was 0.686 (95 % CI 0.630–0.742) and using FN T-score of DXA 0.714 (95 % CI 0.661–0.767). Using only the traditional parameters of DXA (FN T-score), the AUC was 0.706 (95 % CI 0.652–0.760). The AUC for hip osteoporotic fracture was 0.883 (95 % CI 0.827–0.938), 0.857 (95 % CI 0.773–0.941), and 0.814 (95 % CI 0.712–0.916) respectively. For major osteoporotic fractures, the overall predictive value using the ratio Observed fractures/Expected fractures calculated with FRAX without T-score of DXA was 2.29 and for hip fractures 2.28 and with the inclusion of the T-score 2.01 and 1.83 respectively. However, for hip fracture in women < 65 years was 1.53 and 1.24 respectively.

**Conclusions:**

The FRAX tool has been found to show a good discriminatory capacity for detecting women at high risk of fragility fracture, and is better for hip fracture than major fracture. The test of sensibility shows that it is, at least, not inferior than when using BMD model alone. The predictive capacity of FRAX tool needs some adjustment. This capacity is better for hip fracture prediction and better for women < 65 years. Further studies in Catalonia and other regions of Spain are needed to fine tune the FRAX tool’s predictive capability.

## Background

Osteoporosis is of particular public health interest due to its association with subsequent fractures and the well-documented risk of mortality and disability leading to an increase in medical care costs in many regions of the world as a result [1, 2].

Over the last decade the attitude towards osteoporotic fracture risk evaluation has changed because an increase in information about using various clinical risk factors (CRFs) and not only the values of bone mineral density (BMD) [3]. To provide risk assessment, especially for those professionals who are less familiarized with the approach to this health problem, several prediction models have been developed to be used in clinical practice. There are three instruments that have been commonly used in recent times that help to identify people at a high risk of osteoporotic fracture over a period of time: the FRAX® (Fracture Risk Assessment tool) [4], the QFractureScores [5] and the Garvan Fracture Risk Calculator [6, 7].

FRAX instrument was launched by WHO in 2008, and gives the absolute risk of fragility fracture as a percentage during a 10-year period. The risk estimate is carried out by a calculator available online by putting in the value of clinical variables that have shown a strong association with osteoporosis and fracture across different studies and systematic reviews [8–14]. The calculator is able to recalculate the risk with the inclusion of Dual-energy X-ray absorptiometry (DXA) parameters. Epidemiological osteoporotic fracture data in four areas has been used in its construction (clinical spine, distal forearm, hip or proximal humerus), as well as the mortality data available from different continents. Once the tool has been accessed online, it is necessary to select the relevant study population. During the last few years, there have been several studies focused on evaluating how FRAX behaves among different populations other than the one in which the model was developed. Systematic reviews identify studies that assess the FRAX tool ability to discriminate between individuals who are at risk of fracture and those who are not. Also, its predictive ability to identify people at high risk of future fractures, fracture risk thresholds and identifying which risk thresholds are cost-effective when it comes to carrying out a therapeutic intervention. It is still to be determined whether the CRFs are of significance on the outcome of fracture data in different cohorts [15–24].

In Spain, FRAX performance has been assessed during its use in different cohorts since 2008 and it has shown a good discriminative capacity, but a tendency to underestimate major osteoporotic fractures has been observed [25–27]. However, the underestimation was lower in cases of hip osteoporotic fractures. This does not detract from the strengths of the tool for clinical use in decision-making as long as their possible limitations are taken into account.

This study aims to expand the sample before FRIDEX cohort [25] to test the FRAX algorithm against the results of reliability of BMD in its discriminative and predictive ability for predicting absolute risk of fracture in 10 years. This is to provide more evidence for clinicians about how well the tool performs among the Spanish population.

## Methods

The FRIDEX cohort features have previously been published [28]. At the beginning of the study, the participants underwent axial bone densitometry DXA after accepting by informed consent to answer a questionnaire on risk factors (QRF) for osteoporotic fracture and further contact. Self-reported incident fractures 10 years later were assessed using a telephone questionnaire (TQ).

### Eligibility criteria

#### Patient inclusion criteria

Randomized sample (simple computerized randomization stratified) was obtained from Caucasian women ≥ 40 and ≤ 90 years of age at the time of inclusion in the FRIDEX cohort, who understood and spoke the Spanish language, and were able to respond to the initial QRF and a 10-year follow up TQ. None of these patients had been treated with antiosteoporotic medication (AOM) prior to the study. Some of these patients, however, may have been treated with AOM during the 10-year study period.

#### Patient exclusion criteria

Patients who refused informed consent to participate in the study and those without a telephone contact number or did not respond after 3 phone calls made at different times according to the procedure manual. Patients with physical or psychological difficulties that prevented their participation in the study with or whose relatives refused them permission to participate. Subjects with Paget’s disease or bone cancer were also excluded.

#### Data collection

The baseline QRF variables were collected from 2000 to 2010 along with DXA, and they included patient demographic (date of birth, sex) and anthropometric characteristics (weight, height, body mass index (BMI)). During the same visit, clinical risk factors for fracture including QRF were recorded as well: family history of hip fracture (father/mother), medical history of fragility fracture, smoking, alcohol risk intake, history of glucocorticoids intake and medical history of antiosteoporotic medication.

Analysis carried out by using a Lunar GE model *Prodigy Advance* densitometer with 11.4 software and with BMD and T-score determination with NHANES III references. DXA criteria were determined according to the recommendations made by the International Society for Clinical Densitometry (ISCD) in 2007 (http://www.iscd.org/official-positions/). The densitometry diagnostic criteria of osteoporosis used were the 1994 WHO criteria (T-score ≤ −2.5 standard deviation of the average mean value for young women at the femoral neck (FN)) [29].

After a 10-year follow up period (2000–2010) variables regarding new self-reported fragility fractures occurring from the time of inclusion and number of falls over the last year were collected. The major osteoporotic fractures (hip, humerus, forearm and clinical spine) during the follow up period were taken as the endpoint event. In all cases of fracture, medical records were contrasted and those cases of self-reported fractures that were impossible to confirm with medical records were also excluded from analysis.

The estimated absolute risk of sustaining a major osteoporotic fracture or hip fracture during the 10-year period according to the FRAX-Spain tool (both with and without baseline FN T-score) was determined through the official website (version 3.2 accessed on October 2010) and analysed by two blinded investigators.

#### Statistical analysis

The characteristics of the population were described according to descriptive univariate analysis. We used the Chi-square test to evaluate the association between qualitative variables. The Student’s t-test or, if necessary, its nonparametric equivalent, the Mann-Whitney U test, was implemented to evaluate the differences in the distribution of a quantitative variable according to the categories defined by a binary exposure. To assess the differences in the distribution of a quantitative variable according to the categories defined by a categorical variable with more than two categories, ANOVA analysis of variance or its corresponding non parametric test (Kruskal-Wallis) were used.

The discriminating ability of the FRAX tool to identify people at increased risk of fracture after a 10-year period was assessed using the area under the curve (AUC) of receiver operating characteristic (ROC) curves and the Hosmer-Lemeshow goodness-of-fit test.

The calibration was assessed by comparing estimated risk of fracture with observed fracture incidence.

All the statistical tests were undertaken with a confidence interval of 95 % and with the use of the 17th version of the SPSS statistical package.

This work follows the STROBE initiative for cohort studies’ guidelines [http://www.strobe-statement.org/index.php?id=strobe-publications WebCite].

## Results

Three thousand three hundred ninety-seven cohort patients were 2:1 randomly selected among patients who had completed the 10-year period. A total of 1918 women were contacted at the end of the 10-year period and in 1479 cases was impossible to contact by telephone: 490 (14.4 %) unknown telephone or postal address, 792 failed to respond to 3 calls (23.3 %), and 197 deaths (5.8 %). Out of 86 subjects that refused to participate (4.5 %), 33 were excluded due to cancer (1.8 %) and 491 because they had been receiving AOM at baseline (25.6 %). This left a total of 1308 participants that fulfilled the inclusion criteria and provided informed consent to participate in the study.

Table 1 shows the distribution of the baseline characteristics in the individuals selected and those selected, but did not participate in the study. Overall, no significant differences were observed between these two groups. The only significant differences were found among participants with a 2 year age differences (57.5 vs. 59.3 years), that had had fewer previous fractures (22.6 vs 26.0 %), were taking less glucocorticoids (4.7 vs. 6.5 %) and had less osteoporosis according to baseline DXA scan (32.7 vs 37.3 %).

**Table 1:** FRIDEX cohort (Contacted/Non-Contacted)

	Contacted	Non-Contacted	*p*-value	95 % CI
	1918 (56.5 %)	1479 (43.5 %)		
Age (SD)	57.5 (8.1)	59.3 (9.9)	0.001	[1.1–2.4]
BMI Kg/cm^2^ (SD)	27.7 (4.6)	27.5 (4.5)	0.506	ns
BMI < 20 Kg/cm^2^ (%)	2.4	2.6	0.723	ns
Personal history of fractures (%)	22.6	26.0	0.014	[0.5–6.3]
Parental Hip Fracture (%)	22.7	21.1	0.335	ns
Current Smoking (%)	10.8	10.8	0.802	ns
High alcohol intake (%)	0.7	0.9	0.667	ns
Glucocorticoids (%)	4.7	6.5	0.022	[0.2–3.4]
Rheumatoid arthritis (%)	1.3	1.8	0.158	ns
Ca + vitamin D supplements (%)	22.2	22.2	0.916	ns
Osteoporosis in DXA (%)	32.7	37.3	0.012	[1.4–7.8]

*CI* confidence interval, *SD* standard deviation, *BMI* body mass index, *ns* non statistical significance, *DXA* dual-energy X-ray absorptiometry

We examined the frequency of fragility fractures during the 10-year study period: a total of 153 fractures were registered, 133 of which corresponded to any of the four areas of FRAX major osteoporotic fractures: hip, humerus, forearm or clinical spine. 108 women reported a total of 133 major osteoporotic fractures which were contrasted: 26 women with 27 hip fractures, 26 with 33 proximal humerus fractures, 40 with 56 distal radius fractures, and 16 with 20 clinical vertebral fractures (Table 2).

**Table 2:** All fractures at 10 year follow up in two groups of age

	<65 years	≥65 years	Total	*p*-value	95 % CI
	*n* = 1056 (80.7 %)	*n* = 252 (19.3 %)	*n* = 1308		
Age (SD)	54.1 (5.3)	70.3 (4.4)	57.2 (8.2)	<0.001	[15.6–16.9]
	Women/Fractures	Women/Fractures	Women/Fractures		
All fractures	101/129 (9.6 %)	52/74 (20.6 %)	153/203 (11.7 %)	<0.001	[5.7–16.3]
Major fractures^a^	65/78 (6.2 %)	43/55 (17.1 %)	108/133 (8.3 %)	<0.001	[6.0–16.8]
Hip	6/6 (0.6 %)	20/21 (7.9 %)	26/27 (2.0 %)	<0.001	[3.9–10.7]
Vertebral	10/13 (1.0 %)	6/7 (2.8 %)	16/20 (1.4 %)	0.026	[0.3–3.9]
Humerus	21/24 (2.3 %)	5/9 (3.2 %)	26/33 (2.4 %)	0.409	ns
Wrist	28/35 (2.9 %)	12/18 (6.0 %)	40/56 (3.5 %)	0.016	[0.2–6.2]

*CI* confidence interval, *SD* standard deviation, *ns* non statistical significance

^a^Major osteoporotic fractures (hip, vertebra, humerus, wrist)

A summary of the main participants’ characteristics can be seen in Tables 3 and 4 (with their relative risks [RR]). Every risk factor is shown and categorized as major or hip fracture respectively. Risk factors included in the FRAX tool were also taken into consideration, along with the variable of falls during the previous year. The BMD measurement computed by WHO international reference standard for description of osteoporosis as a T-score ≤ −2.5 standard deviation (SD) and osteopenia as a T-score between −1.0 and −2.5 SD was also taken into account. The CRFs values across both major and hip fractures show significant differences in age, previous fractures and due to the existence of DXA osteoporosis diagnosis. Table 3 shows in the analysis of major osteoporotic fracture significant differences in patients with fractures related to low BMI and in patients without fractures related to normal baseline DXA. Table 4 shows significant differences among patients with rheumatoid arthritis and taking corticoids in the hip osteoporotic fracture group.

**Table 3:** Baseline fracture risk factors between patients for ‘major osteoporotic fracture’

	With Fracture (*n* = 108)	Without Fracture (*n* = 1200)	*p*- value	95 % CI	RR	RR 95 % CI
Age (SD)	61.6 (9.4)	56.8 (8.0)	<0.001	[2.9–6.6]		
Age > 65 years (%)	39.8	17.4	<0.001	[12.9–31.9]	2.77 ^a^	[1.9–4.0]
BMI (SD)	27.6 (4.6)	28.0 (4.7)	0.518	ns	-	-
BMI < 20 (%)	5.6	2.3	0.036	[1.1–7.7]	2.27 ^b^	[1.1–4.4]
Previous fracture (%)	42.6	19.5	<0.001	[13.5–32.7]	2.72	[1.9–3.9]
Parental hip fracture (%)	15.7	14.1	0.637	ns	1.13	[0.7–1.8]
Smoker (%)	9.3	11.7	0.452	ns	0.79	[0.4–1.4]
Alcohol risk (%)	0.9	0.7	0.541	ns	1.35	[0.2–5.4]
Corticoids (%)	8.3	4.7	0.093	ns	1.74	[0.9–3.2]
Rheumatoid arthritis (%)	2.8	1.0	0.120	ns	2.46	[0.9–5.7]
Falls previous year (%)	34.3	22.3	0.005	[2.7–21.3]	1.71	[1.2–2.5]
Osteoporosis (baseline DXA) (%)	51.9	26.1	<0.001	[16.1–33.6]	6.07 ^c^	[2.9–12.9]
Osteopenia (baseline DXA) (%)	41.7	49.9	0.103	ns	2.95 ^d^	[1.4–6.4]
Normal (baseline DXA) (%)	6.5	24.0	<0.001	[12.3–22.7]	-	-

*CI* confidence interval, *RR* relative risk, *SD* standard deviation, *BMI* body mass index, *ns* non statistical significance, *DXA* dual-energy X-ray absorptiometry

^a^ < 65 vs ≥ 65 years

^b^ < 20 vs ≥ 20

^c^ Osteoporosis vs normal

^d^ Osteopenia vs normal

**Table 4:** Baseline fracture risk factors between patients for ‘hip osteoporotic fracture’

	With fracture (*n* = 26)	Without fracture (*n* = 1282)	*p*- value	95 % CI	RR	RR 95 % CI
Age (SD)	69.7 (6.8)	56.9 (8.0)	<0.001	[9.7–15.9]	-	-
Age > 65 (%)	76.9	18.1	<0.001	[42.5–75.1]	13.97 ^a^	[5.8–33.5]
BMI (SD)	27.2 (3.5)	27.9 (4.8)	0.310	ns		
BMI < 20 (%)	3.8	2.5	0.664	ns	1.55 ^b^	[0.3–8.2]
Previous fracture (%)	53.8	20.7	<0.001	[13.8–52.4]	4.28	[2.0–9.0]
Parental hip fracture (%)	19.2	14.1	0.460	ns	1.44	[0.6–3.6]
Smoker (%)	0	11.7	0.063	ns	-	-
Alcohol risk (%)	0	0.7	0.834	ns	-	-
Corticoids (%)	15.4	4.8	0.036	[3.3–24.5]	3.48	[1.3–9.2]
Rheumatoid arthritis (%)	7.7	1.0	0.034	[3.6–17.0]	7.18	[1.9–22.3]
Falls previous year (%)	38.5	23.0	0.065	ns	2.06	[1.0–4.4]
Osteoporosis (baseline DXA) (%)	65.4	27.5	<0.001	[19.4–56.3]	6.80 ^c^	[1.8–26.3]
Osteopenia (baseline DXA) (%)	26.9	49.7	0.021	[5.5–40.1]	1.60 ^d^	[0.4–6.8]
Normal (baseline DXA) (%)	7.7	22.9	0.067	ns	-	-

*CI* confidence interval, *RR* relative risk, *SD* standard deviation, *BMI* body mass index, *ns* non statistical significance, *DXA* dual-energy X-ray absorptiometry

^a^ < 65 vs ≥ 65 years

^b^ < 20 vs ≥ 20

^c^ Osteoporosis vs normal

^d^ Osteopenia vs normal

The mean of FRAX risk for major fracture among women with fracture was 6.44 (6.94 SD) without FN T-score and 8.25 (9.19 SD) with FN T-score, and for hip fracture it was 2.38 (5.20 SD) and 3.59 (7.39 SD), respectively. The mean for major fracture among without fracture women was 3.35 (2.81 SD) without FN T-score and 3.73 (3.48 SD) with FN T-score, and for hip fracture it was 0.74 (1.40 SD) and 0.86 (1.94 SD), respectively. All measurements show significant differences (*p* < 0.001) between women with fracture and without fracture.

The AUC ROC analysis was carried out to compare fracture discrimination on the basis of three different scenarios for major and hip fractures: guidance on the basis of BMD testing alone in decision-making (FN T-score), FRAX tool calculated without BMD, and FRAX with FN T-score. For major fracture the best-case scenario was obtained with FRAX tool including FN T-score [AUC = 0.714, 95 % CI 0.661–0.767], followed by FN BMD alone [AUC = 0.706, 95 % CI 0.652–0.760] and FRAX tool without BMD [AUC = 0.686, 95 % CI 0.630–0.742]. For hip fracture the best-case performance analysis was obtained with FRAX without BMD [AUC = 0.883, 95 % CI 0.827–0.938], followed by FRAX including FN T-score [AUC = 0.857, 95 % CI 0.773–0.941] and FN BMD alone [AUC = 0.814, 95 % CI 0.712–0.916]. In all cases, the results showed significant differences (*p* < 0.001) with the reference value [AUC = 0.50].

The adjusted predictive capacity of FRAX analysed using the mean ratio between observed fractures (ObsFx) during the 10-year follow-up period of the cohort and the fracture risk estimates rates (ExpFx) was 2.29, CI 95 % 1.91–2.74) for major osteoporotic fracture and 2.28 [CI 95 % 1.56–3.32] for hip fracture using the FRAX tool without BMD, and on the introduction of the FN T-score was 2.01 [CI 95 % 1.68–2.41] and 1.83 [CI 95 % 1.25–2.67], respectively (Table 5). This ratio remained similar when we categorized the results based on age, except among women younger than 65 years of age in which case the FRAX without/with BMD result dropped in the hip fracture category to 1.53 [CI 95 % 0.70–3.32] and 1.24 [CI 95 % 0.57–2.68], respectively.

**Table 5:** Ratio Observed fractures/Expected fractures by FRAX tool by age

	Major fractures^a^				Hip fractures			
All (1308 women)	Obs Fx	Exp Fx	Ratio Obs/Exp	CI 95 %	Obs Fx	Exp Fx	Ratio Obs/Exp	CI 95 %
FRAX tool without BMD	108	47.1	2.29	[1.9–2.4]	26	11.41	2.28	[1.6–3.3]
FRAX tool with T-score FN	108	53.6	2.01	[1.7–2.4]	26	14.20	1.83	[1.3–2.7]
<65 years (1056 women)	Obs Fx	Exp Fx	Ratio Obs/Exp	CI 95 %	Obs Fx	Exp Fx	Ratio Obs/Exp	CI 95 %
FRAX tool without BMD	65	27.2	2.39	[1.9–3.0]	6	3.91	1.53	[0.7–3.3]
FRAX tool with T-score FN	65	30.6	2.12	[1.7–2.7]	6	4.84	1.24	[0.6–2.7]
≥65 years (252 women)	Obs Fx	Exp Fx	Ratio Obs/Exp	CI 95 %	Obs Fx	Exp Fx	Ratio Obs/Exp	CI 95 %
FRAX tool without BMD	43	19.9	2.16	[1.6–2.8]	20	7.50	2.67	[1.7–4.0]
FRAX tool with T-score FN	43	23.0	1.87	[1.4–2.4]	20	9.36	2.14	[1.3–3.2]

*ObsFx* observed fractures, *ExpFx* expected fractures, *CI* confident interval, *BMD* bone mineral density, *FN* femoral neck

^a^Major fractures (hip, vertebra, humerus, wrist)

**Fig. 1:**
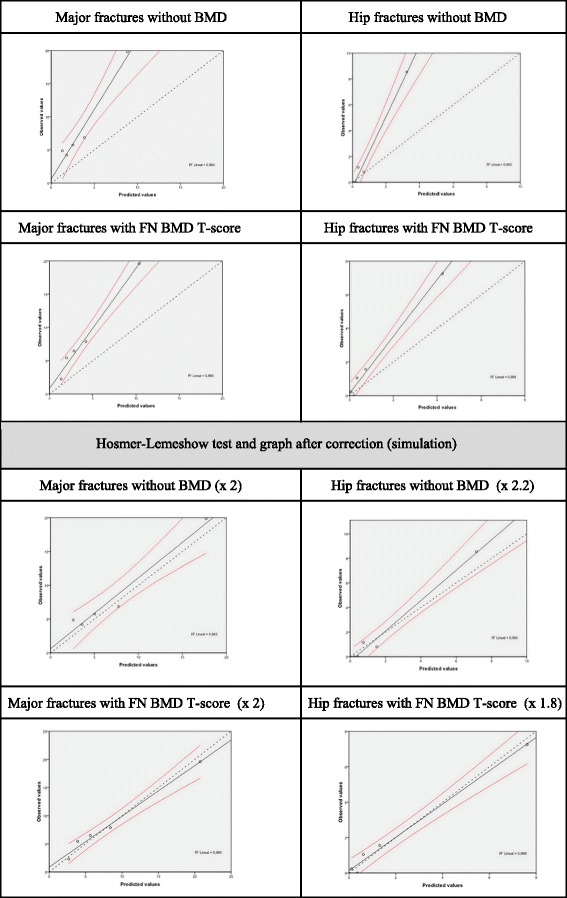
Hosmer-Lemeshow test with the original results of the FRAX tool

The Hosmer-Lemeshow test was carried out in order to assess the ‘goodness of fit’ obtained by grouping data according to quintiles of results of fracture (Fig. 1). First of all it shows the observed and predicted values of the sample within major fracture and hip fracture for the results of the FRAX tool without BMD and with the FN BMD T-score. It then shows the same results after multiplication (simulation) by approximately the number of times that the ObsFx is greater than the ExpFx.

## Discussion

The FRAX tool has been analysed in this study to measure its discriminative capacity as a model for the prediction of osteoporotic fracture compared with the BMD model, as well as its predictive capacity and the ‘goodness of fit’ among the Spanish female population. A previous calibration test as an evaluation of the reliability assessment of FRIDEX cohort results was carried out using a lower number of women [25]. This analysis suggests that the results are consistent with the above-mentioned population. This study also provides information on the frequency of risk factors of osteoporotic fractures.

### Risk factors of osteoporotic fracture

Age is a variable related to the incidence of fracture and in our cohort the overall reported rates of fractures is higher in the group over 65 years old (*p* < 0.001). When focusing individually on hip, clinical spine, forearm and humerus fractures, the proportion of fractures increases with age. However, these differences were not statistically significant in the case of humerus fractures in our sample. In addition to age, prior fragility fracture, low BMI, rheumatoid arthritis or glucocorticoids intake are clinical factors related to the pathogenesis of osteoporotic fracture, and indeed, the results of our study have shown statistical significance in major fracture and hip fracture. These results align well with other studies undertaken in our population, which have identified the same relevant factors for fragility fracture except for BMI [30]. We also have found previously published data that investigate relationship between BMI and fracture in Spanish postmenopausal women, but focusing in high BMI. In this context, some reported a relation between vertebral fracture and high BMI [31] and, conversely, other studies found no relation in this site, only for proximal humerus fractures [32]. So further studies will be needed to clarify these variations.

We also note that in evaluating Spanish FRAX tool estimates without T-score, the risk of main fracture and hip fracture obtained is significantly higher among women with fractures than those without fractures. The contribution of BMD in the osteoporotic fracture risk is reflected in the results of our sample as well, showing a lower average of BMD and increased FRAX estimates with FN T-score between women with fracture compared to women without fracture (*p* <0.05) (Tables 3 and 4).

### Assessing FRAX-Spain discrimination performance

The ROC analysis used to assess the discriminatory capacity of the FRAX osteoporotic fracture estimates, shows a more accurate AUC of FRAX in major fractures with a FN T-score [0.714, 95 % CI 0.661–0.767] and for hip fracture with FRAX without BMD [0.883, 95 % CI 0.827–0.938)]. These findings match those already reported in the previous FRIDEX sample and are similar to those found in other research conducted in Spain [25, 27].

The aforementioned finding bear out the current trend for assessing fracture risk using clinical risk factors rather than only using the DXA results.

Regarding the older population with a higher susceptibility to fractures, especially in the case of hip fractures, these results are particularly relevant as they enhance clinical decision-making in practices that have a more limited access to DXA. It is worth noting that results in other nearby countries have shown a similar ability using FRAX with FN T-score to identify women at a high risk of major fracture compared to when the FN BMD is solely used [15, 19].

### Assessing FRAX-Spain predictive performance

The adjusted predictive capacity of the FRAX tool analysed using the ObsFx/ExpFx ratio shows no correlation between observed and expected fracture rates among Spanish population. All the women in the sample cohort when analysed together showed a higher frequency of fragility fracture (close 2 times more) than would be expected with FRAX tool either if it is calculated with the FN T-score or without BMD (Table 5). This difference is lower for hip fracture with FRAX with FN T-score of DXA (ObsFx/ExpFx ratio all cohort 1.83) and clearly better in women < 65 years old (Table 5). It could be explained by the mean age of the women in the study, with 80 % of the cohort being under the age of 65. Osteoporotic fracture that tends to occur in early menopause affects the lumbar spine more than the hip, as hip BMD decreases exponentially with advancing age [33–35]. As observed in our study, when the ratio of observed to expected hip fracture is calculated for those aged 65 or older with FRAX with FN T-score, the probability of hip fracture increases two fold again.

The Hosmer-Lemeshow test was carried out to assess the goodness of fit between observed fractures and the expected fractures according to FRAX. A satisfactory goodness of fit was obtained by multiplying the results by the ObsFx/ExpFx ratio taking into account the CI 95 % (Fig. 1).

The Spanish FRAX model has been evaluated in other cohorts. The ECOSAP cohort published similar hip fracture risk prediction, but they did not collect clinical vertebral fractures, therefore the interpretation of the results for prediction of major osteoporotic fractures is difficult [26]. In contrast, the methodology for collecting incident fractures considered by FRAX in the CETIR database was complete (clinical spine, hip, distal forearm and proximal humerus) and self-reported with further validation too [27]. In the results observed, the ObsFx/ExpFx ratio for major fractures was 2.4 (CI 95 %: 2.1–2.7) and 0.8 (CI 95 %: 0.6–1.1) for hip fractures. Therefore, the major fracture results are in accordance with our findings indicating that the FRAX model underestimates fracture risk in Spanish women [25]. The possible explanation for this underdiagnosed has already been justified because for Spain, the data included in FRAX are from studies conducted in the 90s [25–27], validated in areas of a low incidence of hip fracture and more up-to-date fracture incidence and mortality data is required for fracture predictions [18, 36–38]. Therefore, some authors have suggested that these methodological factors may affect the interpretation of calibration, and should be taken into account before making an assessment of the tool [39–41]. The ratio for hip fracture in this case is closer to 1, the desired value. One possible explanation of this lack of accuracy may be due to the fact that, although the initial formation of the two cohorts followed very similar schemes, the other female Spanish cohorts were younger [25, 27]. The differences among the three Spanish cohorts’ findings can also be justified by the fact that the ECOSAP and CETIR cohorts were comprised of a low proportion of women over 70 years, a shorter average follow-up period, a low proportion of hip fractures and a different method of follow up was used [26, 27]. Therefore, this could account for the differences in the hip fracture ratio. The predicted probabilities of fragility fracture using the Spanish FRAX tool have also been analysed in FRODOS and ESOSVAL cohorts but the observed incidence of osteoporotic fracture was not recorded. Therefore, their data cannot be used to assess the predictive ability of the tool [42, 43].

The study has some strengths and limitations. The strengths of our study include among the 3397 potentially eligible subjects contacted for the study, there were no significant differences in most of the basic characteristics between participants and non-participants. The differences found in mean age, prior fracture, corticosteroids use and osteoporosis BMD result were very small, thus we assume that the sample was representative from the population from which it was taken. To determine incident osteoporotic fractures some countries, in the first place, review hospital hip fracture discharge statistics assuming that all proximal femoral fractures result in hospitalization. In the case of the remaining major fractures (humerus, clinical spine, forearm) others studies used incidence fracture data taken from a cohort in Malmö, working on the premise that the ratios would be similar [44–48]. In the present study, all fractures recorded via the use of a telephone questionnaire were contrasted with existing medical record data and only included in the final analysis if the fractures were also found both in the medical records and via the telephone questionnaire. There are also limitations. Working on the assumption that the women included in the FRIDEX cohort could have a higher risk of osteoporotic fractures than the general population due to the fact that it is a population that had previously been selected to undergo a DXA scan for different reasons. Currently, there is evidence that the women included in the FRIDEX cohort have not a higher incidence of fragility fracture than the general population, although they have more risk factors for fragility fracture [7, 25, 49]. In the present analysis, deceased patients were excluded and this should be taken into consideration. Despite the fact the number of deceased patients only made up 5.8 % of the cohort, it could lead to a misinterpretation of observed fractures [50]. Finally, our data has been confined to women and we still do not know if a similar result would be obtained in men, further studies would be necessary to ascertain this.

## Conclusions

In summary, based on the study’s finding, the FRAX tool has been found to show a good discriminatory capacity for detecting women at high risk of fragility fracture. In the case of hip fractures, this discriminatory capacity of the FRAX tool without BMD was found to be higher than when using BMD alone. Possibly, further studies in Catalonia and other regions of Spain would be required to fine tune the FRAX tool’s predictive capability.

## Abbreviations

AOM, antiosteoporotic medication; AUC, area under curve; BMD, bone mineral density; BMI, body Mass Index; CI, confidence Interval; CRFs, clinical risk factors; DXA, dual-energy X-ray absorptiometry; ExpFx, expected fractures; FN, femoral neck; ISCD, International Society for Clinical Densitometry; ObsFx, observed fractures; QRF, questionnaire on risk factors; ROC, receiver operating characteristic; RR, relative risk; SD, standard deviation; TQ, telephone questionnaire
